# Supplementary material for: Epidemiology and transmission of hepatitis A in Shaanxi (western China) after more than ten years of universal vaccination
Source: PeerJ. 2023 Nov 13;11:e16305. doi: 10.7717/peerj.16305 (PMC10652842; doi:10.7717/peerj.16305)
Supplement: Supplemental Information 3 — # sample size was calculated by following equator (N = P*(1-P)*z2/MOE2), then this number was multiplied by 1.2 , in which z was type I error (α = 0.05), P was prevalence, and MOE was precision (confidence interval/2). Prevalence of Hepatitis A was estimated based on previous seroprevalence study, considering the efficiency of cluster sampling, the sample size was multiplied by 1.2 in the final version. [file peerj-11-16305-s003.docx]

Supplement table 3

| Birth year | Age group | Historical data* | Sample size # |
| --- | --- | --- | --- |
| 2009-2016 | 2-9 | 77.46% | 328 |
| 2003-2008 | 10-15 | 66.69% | 411 |
| 1992-2002 | 16-27 | 69.24% | 395 |
| 1980-1991 | 28-38 | 80.53% | 291 |
| 1958-1979 | 39-60 | 94.94% | 90 |
| total |  |  | 1508 |

*this data was retrieved from published articles (Kong et al., 2017, Yan et al., 2019)

# sample size was calculated by following equator (N=P*(1-P)*z^2^/MOE^2^), then this number was multiplied by 1.2, in which z was type I error (α=0.05), P was prevalence, and MOE was precision (confidence interval/2). Prevalence of Hepatitis A was estimated based on previous seroprevalence study, considering the efficiency of cluster sampling, the sample size was multiplied by 1.2 in the final version

Reference

Kong Q, Yan BY, Lyu JJ, Feng Y, Liu JY, Song LZ, et al. [Prevalence of hepatitis A antibody among population covered by different hepatitis A immunization strategies in Shandong Province, 2015, China]. Zhonghua Yu Fang Yi Xue Za Zhi 2017;51(6):480-3.

Yan BY, Lv JJ, Liu JY, Feng Y, Wu WL, Xu AQ, et al. Changes in seroprevalence of hepatitis A after the implementation of universal children vaccination in Shandong Province, China: a comparison between 2006 and 2014. International journal of infectious diseases : IJID : official publication of the International Society for Infectious Diseases 2019.
